# On-Surface Synthesis of Nanographenes and Graphene Nanoribbons on Titanium Dioxide

**DOI:** 10.1021/acsnano.2c10416

**Published:** 2023-01-24

**Authors:** Rafal Zuzak, Jesus Castro-Esteban, Mads Engelund, Dolores Pérez, Diego Peña, Szymon Godlewski

**Affiliations:** † Centre for Nanometer-Scale Science and Advanced Materials, NANOSAM, Faculty of Physics, Astronomy and Applied Computer Science, Jagiellonian University, Łojasiewicza 11, PL 30-348 Krakow, Poland; ‡ Centro de Investigación en Química Biolóxica e Materiais Moleculares (CiQUS) and Departamento de Química Orgánica, Universidade de Santiago de Compostela, 15782 Santiago de Compostela, Spain; § Espeem S.A.R.L. (espeem.com), 12 Cité Franz Leesbierg, L-4206 Esch-sur-Alzette, Luxembourg

**Keywords:** titanium dioxide, nanographene, graphene nanoribbon, on-surface synthesis, cyclodehydrogenation

## Abstract

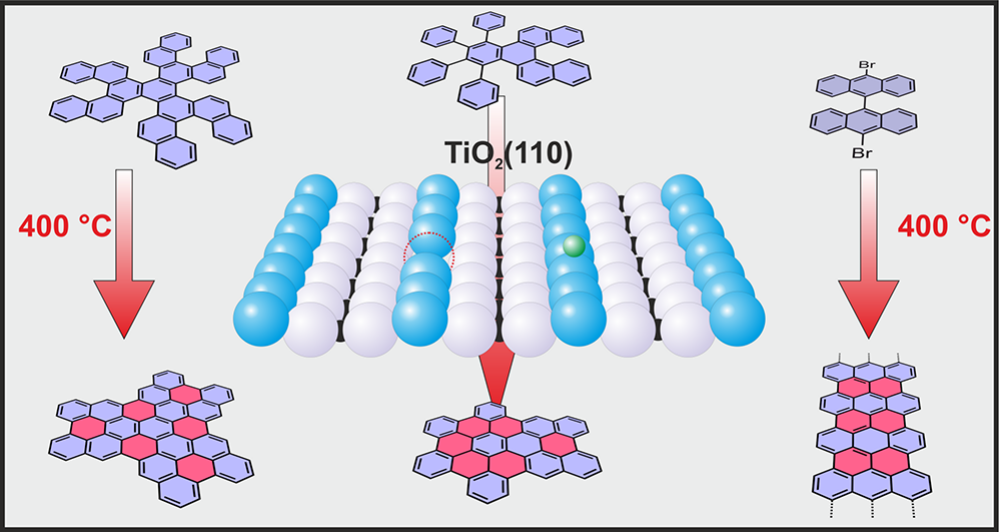

The formation of two types of nanographenes from custom designed and synthesized molecular precursors has been achieved through thermally induced intramolecular cyclodehydrogenation reactions on the semiconducting TiO_2_(110)-(1×1) surface, confirmed by the combination of high-resolution scanning tunneling microscopy (STM) and spectroscopy (STS) measurements, and corroborated by theoretical modeling. The application of this protocol on differently shaped molecular precursors demonstrates the ability to induce a highly efficient planarization reaction both within strained pentahelicenes as well as between vicinal phenyl rings. Additionally, by the combination of successive Ullmann-type polymerization and cyclodehydrogenation reactions, the archetypic 7-armchair graphene nanoribbons (7-AGNRs) have also been fabricated on the titanium dioxide surface from the standard 10,10′-dibromo-9,9′-bianthryl (DBBA) molecular precursors. These examples of the effective cyclodehydrogenative planarization processes provide perspectives for the rational design and synthesis of molecular nanostructures on semiconductors.

The atomically precise synthesis of custom designed molecules as well as more extended nanostructures is one of the promising approaches for the development of functional materials that can be used in more efficient electronic devices in the future. In recent years, it has been demonstrated that a wide range of molecular nanostructures including e.g., nanographenes,^1−10^ graphene nanoribbons (GNRs),^11−20^ porous graphene,^21,22^ nonbenzenoid carbon networks,^23^ and elusive intrinsically instable compounds^24−31^ could be fabricated through the surface-assisted synthesis methods. This bottom-up approach is based on the generation of target structures from air-stable molecular precursors, which are transformed into the final products through chemical reactions initiated on surfaces usually in ultrahigh vacuum conditions. While the approach has been very successful in the generation of molecular architectures, which have not been achieved based on traditional solution chemistry, one of its main drawbacks is the strong reliance on the catalytic activity of the substrates. This significantly limits the class of suitable materials which could be used as a substrate to mainly noble metals, leaving the issue of surface-assisted synthesis on nonmetallic materials, hence technologically desired surfaces, generally unsolved. Up to now, the reports on the on-surface synthesis on semiconductors and insulators are rare^32^ including dehalogenative polymerization on certain surfaces,^33−36^ light-induced coupling on KCl,^37^ on mica or silicon oxide,^38^ and on hexagonal boron nitride,^39^ and intermolecular coupling on calcite^40−45^ to name a few. Nevertheless, there is the general inability to carry out cyclodehydrogenation processes, which are the main path toward planarization and aromatization forces to search for alternative scenarios. For instance, recently, Kolmer et al. reported an approach based on application of the cyclodehydrodefluorination reaction, which could be initiated on the (011) face of rutile titanium dioxide providing partially planarized nanostructures.^46^ However, such a method requires the use of specially designed precursors, which are difficult to synthesize, as well as the application of tip induced reactions in order to create more extended planarized units.^47^ These constraints make the approach highly specific and local.

Here, we demonstrate that well-shaped large nanographenes as well as GNRs could be efficiently synthesized on the most relevant and stable face of TiO_2_, namely the (110) surface through thermally induced cyclodehydrogenation (Figure 1). Our experiments indicate that the approach is universal and provides a pathway toward cyclodehydrogenative synthesis of graphene-based nanostructures from a wide range of molecular precursors on TiO_2_(110). Additionally, high-resolution scanning tunneling microscopy (STM) imaging combined with single point scanning tunneling spectroscopy (STS) and theoretical modeling indicates that the filled-state frontier orbitals of the synthesized nanographenes are not strongly influenced by the presence of the substrate.

**Figure 1:**
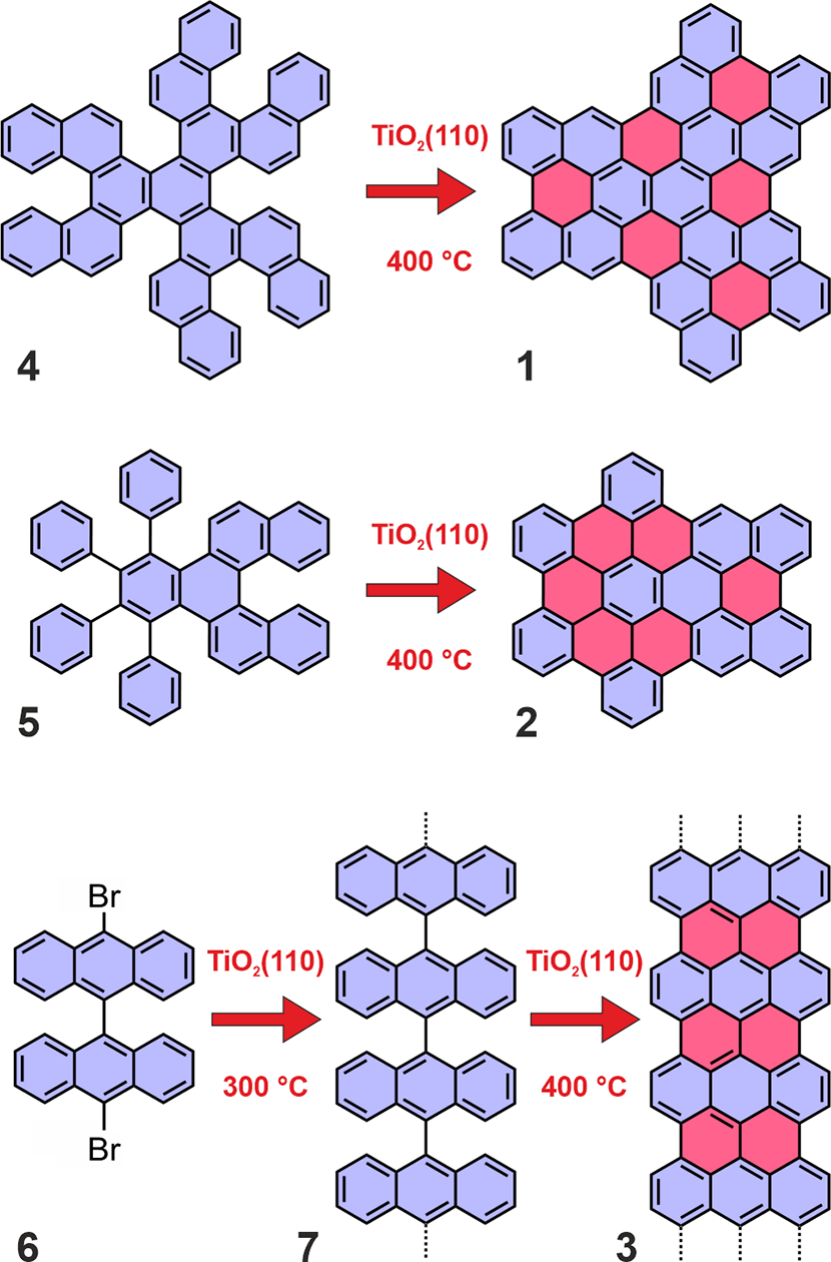
Schematic illustration of synthetic paths providing nanographenes **1** and **2**, 7-AGNR (**3**), and DBBA-based polymers (**7**) on TiO_2_(110)-(1×1) from the corresponding molecular precursors (i.e., **4**, **5**, and **6**). The red color represents additional six-membered rings fabricated in the on-surface experiments.

## Results and Discussion

### On-Surface Synthesis of Nanographenes

For our experiments, we have chosen two specially designed precursors **4** and **5**, as well as the popular 10,10′-dibromo-9,9′-bianthracene (DBBA, **6**) for the synthesis of 7-AGNR (**3**). The structure of target nanographenes **1** and **2**, as well as the nanographene precursors **4** and **5**, is schematically visualized in Figure 1. Compound **4** is a polycyclic aromatic compound containing six pentahelicene moieties incorporated into its structure, and it has been previously used by us for the synthesis of nanographene **1** on Au(111).^3^ Compound **5** has one pentahelicene unit embedded and is equipped with four side phenyl substituents. Our choice of the starting material is motivated by the striving to demonstrate the wide applicability and versatility of the cyclodehydrogenative approach with diverse structural arrangement of the starting material. The inclusion of the pentahelicene moieties in **4** generates strain due to steric interactions, which cannot be completely relieved within the polycyclic compound. This may influence the ability and conditions for the intramolecular cyclodehydrogenation. In contrast, the free rotation around the σ bond of phenyl rings attached to an aromatic core would allow for more efficient strain relief. Our control experiment on Au(111) revealed that nanographene **1** could be synthesized from compound **4** already at 180 °C in a similar temperature range as e.g. peripentacene.^4,5^ However, the generation of additional benzene rings by fusion of neighboring phenyl rings requires on Au(111) a much higher annealing temperature.^2,11,48^ Therefore, we have incorporated four phenyl rings attached to a benzopentahelicene core into compound **5** to analyze the applicability of the controlled cyclodehydrogenation on TiO_2_(110) in differently shaped precursors.

**Figure 2:**
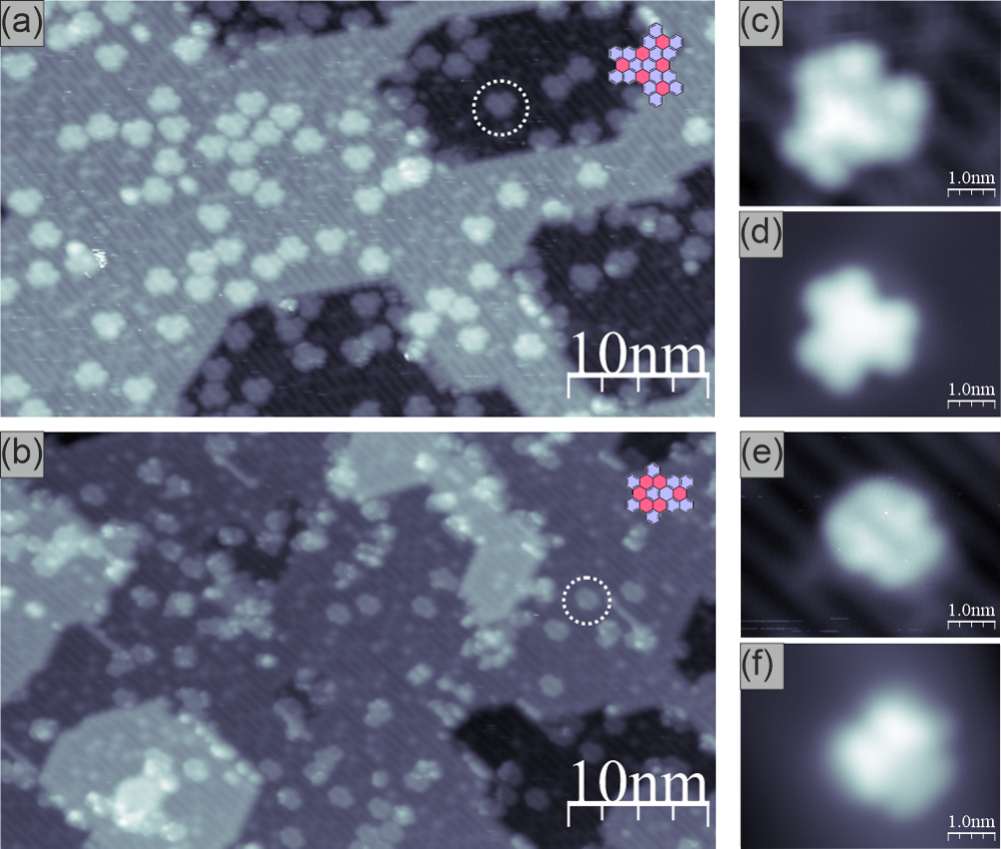
Empty-state STM images of nanographenes **1** (a) and **2** (b) synthesized through on-surface cyclodehydrogenation on the TiO_2_(110) surface. Dashed white circles mark exemplary nanographenes. High-resolution STM images of nanographene **1** on (c) TiO_2_(110) and (d) Au(111) and nanographene **2** on (e) TiO_2_(110) and (f) Au(111); bias voltage: +1.2 V (a, b, c, e); −1.0 V (d, f); tunneling current: 25 pA.

First, we observe that the deposition at room temperature of both **4** and **5** on TiO_2_(110) does not lead to any ordered molecular assemblies. In both cases, annealing of the TiO_2_(110) sample at 400 °C results in formation of planar nanostructures. The shape of recorded STM images is consistent with the expected appearance of nanographenes **1** and **2**, as could be clearly inferred from Figure 2a,b, as well as the magnified images displayed for nanographenes **1** (Figure 2c) and **2** (Figure 2e). Both nanographenes **1** and **2** have also been synthesized for comparison on Au(111) by thermally driven cyclodehydrogenation at 210 and 390 °C, respectively. The corresponding images are shown in Figure 2d,f. These observations suggest the successful transformation of both precursors **4** and **5** into planar and well-shaped nanographenes **1** and **2**, respectively.

**Figure 3:**
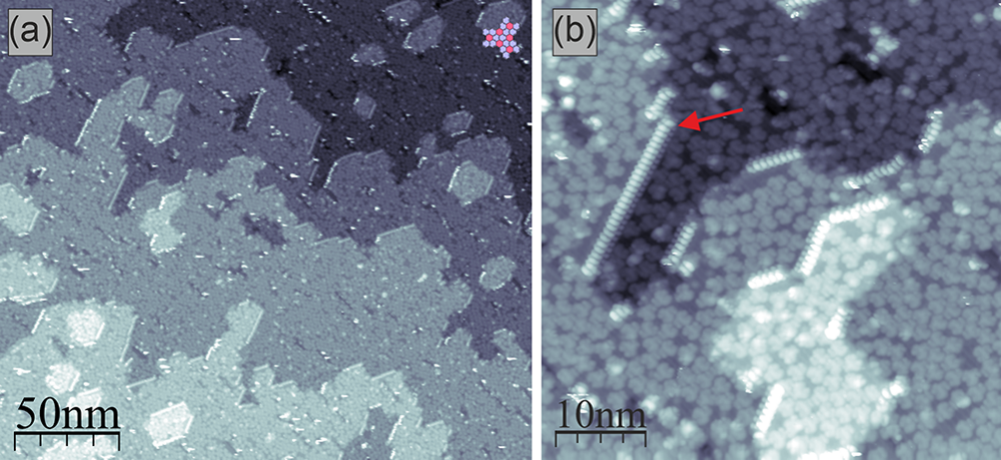
Empty-state STM images of a full layer of nanographenes **1** on TiO_2_(110): (a) overview large scale image and (b) high-resolution image with clearly discernible single nanographene **1**. The red arrow indicates the molecules up-right oriented at the surface step edge; bias voltage: +1.2 V; tunneling current: 25 pA.

Detailed analysis of STM images corresponding to nanographene **1** leads to the conclusion that more than 99% of precursor **4** could be identified as transformed into target compound **1** (in fact, there are only single species that may not correspond to fully converted precursors; see Supporting Information section 1.5.1 for details). Therefore, we have attempted to produce a full layer of flat-lying nanoflakes **1**. The results are shown in Figure 3 visualizing the TiO_2_(110) crystal completely covered by nanographenes **1**. The experiment demonstrates that precursor **4** does not desorb from the surface when heated up to the required temperature of 400 °C.

The situation is different in the case of precursor **5**. The analysis of the images recorded for the samples annealed at different temperatures (see Supporting Information section 1.5.2 for details) leads to the conclusions that at 400 °C - due to desorption - less than 25% of initially deposited molecules could be still found on the surface. From these, a detailed analysis of the species present on the surface enables efficiency estimation for the conversion **5** → **2** process. Approximately 60% (±10%) of the compounds could be classified as nanographene **2** (see Supporting Information section 1.5.2 for details). These observations indicate that the limiting factor for the planarization is the molecule desorption. In the case of precursor **4**, the binding energy of the extended polycyclic system with the underlying substrate is sufficient to reach the required annealing temperature to allow planarization without initiating desorption processes, while the smaller compound **5** does not provide a sufficient energy barrier to separate the desorption from cyclodehydrogenation processes resulting in limited efficiency. In comparison, the cyclization could not be achieved e.g. for hexaphenylbenzene precursors, as these desorb from the surface precluding from formation of hexabenzocoronenes (for details see Supporting Information section 1.6). However, in the case of precursor **5**, the presence of a single pentahelicene unit increases the interaction with the surface and allows for successful cyclodehydrogenation within a fraction of initially deposited precursors. Therefore, we could conclude that in order to successfully initiate and complete the cyclodehydrogenation processes it is compulsory to prepare precursors that would remain on the surface when annealing at the temperatures up to 400 °C.

### Scanning Tunneling Spectroscopy

**Figure 4:**
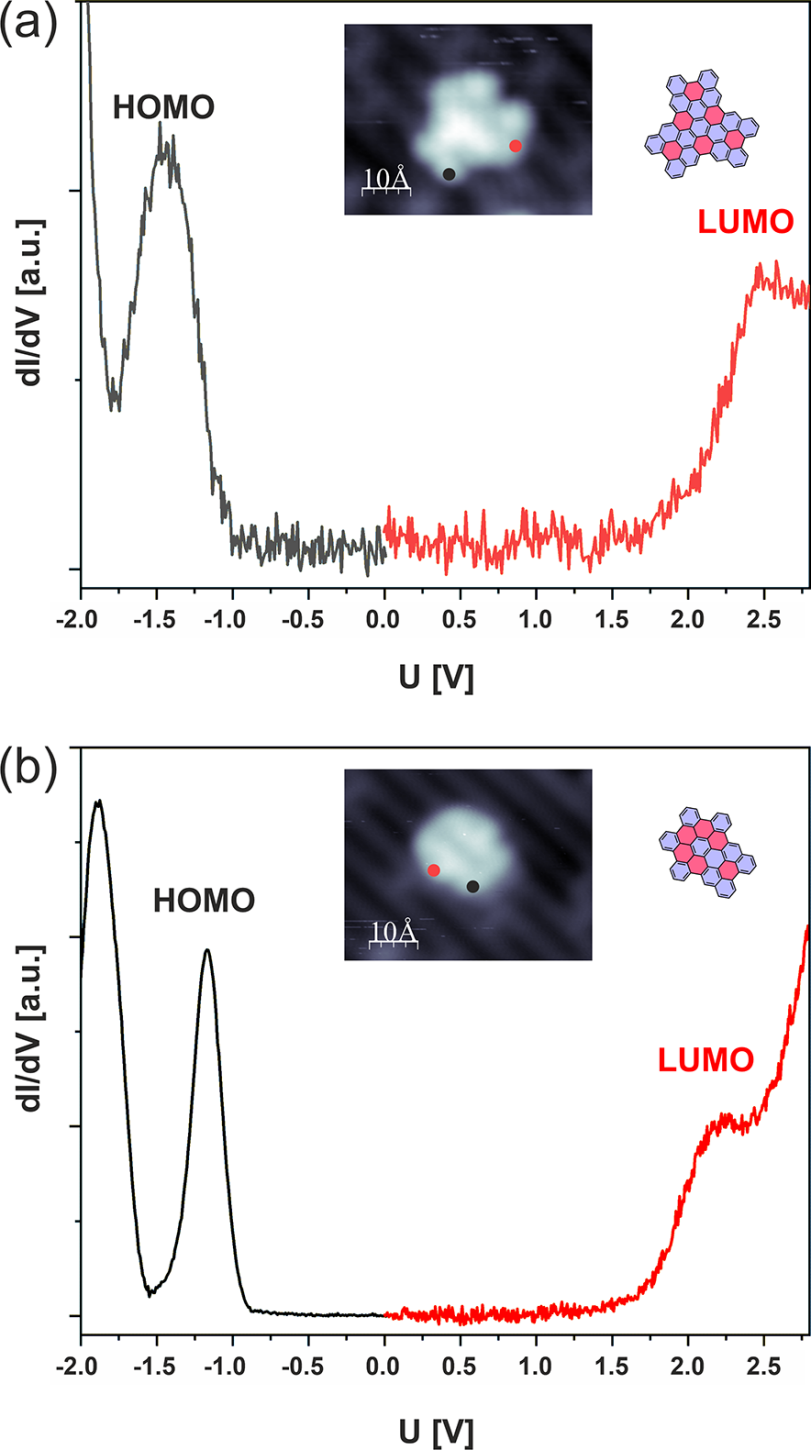
Single point STS spectra recorded for nanographenes **1** (a) and **2** (b) on the TiO_2_(110) surface. The insets show structural models and the STM images of the nanographenes recorded with the voltages corresponding to the molecule band gap (i.e., +1.2 V). Black and red dots in the insets indicate the lateral position of the STM tip during STS data acquisition.

To convincingly demonstrate the fabrication of target compounds **1** and **2**, we have applied high-resolution imaging corroborated with scanning tunneling spectroscopy (STS) and theoretical modeling. At first, we have recorded the single point STS data in order to determine the energies of resonances corresponding to electronic levels of the nanographenes **1** and **2**. The data is visualized in Figure 4a,b. The recorded curves clearly show the presence of pronounced resonances within the filled-state part of the spectra and fainter, broader ones in the empty-state regime. We assume here that these resonances arise from the HOMO (highest occupied molecular orbital) and LUMO (lowest unoccupied molecular orbital) states of the molecules. For **2**, the recorded resonances are located at approximately −1.15 V and +2.25 V, which correspond to the STS measured transport gap of 3.4 eV (Figure 4b). For **1**, the resonances are centered at approximately −1.45 V and +2.45 V, and the resulting STS transport gap reaches 3.9 eV (Figure 4a). The latter value could be compared with the results obtained previously for **1** on Au(111), where the STS measured gap was estimated at approximately 2.2 eV. The discrepancy between the results obtained on Au(111) and TiO_2_(110) could be understood if we take into account the gap renormalization effects on metal-molecule interfaces, which lead to shrinkage of the HOMO–LUMO energy separation.^49,50^ On the other hand, we may also encounter the increase of the energy separation between STS HOMO/LUMO resonances for molecules physisorbed on semiconductors, e.g. due to the band bending effects.^51,52^

The shape of the recorded resonances is reasonable for molecules located on the reduced TiO_2_(110) surface obtained in a standard preparation procedure of consecutive ion bombardment and subsequent annealing processes.^34^ In such a case, the conduction band onset of the substrate shall be located much closer to the Fermi level than the valence band edge.^53^ Sánchez-Sánchez et al. estimated the position of the theoretical Fermi level approximately 0.5 eV below the conduction band onset.^53^ Contrary, the rapidly growing density of states for the valence band appears only 2 eV below the Fermi level. Therefore, we may expect the STS molecular resonances to be pronounced in the filled-state range because of the strongly limited density of substrate states and consequently weak interaction and broadening. Conversely, for molecular empty states, stronger interaction with the substrate may occur, as the molecular resonances are captured in the range of the onset of the substrate conduction band.

### High-Resolution STM Imaging

**Figure 5:**
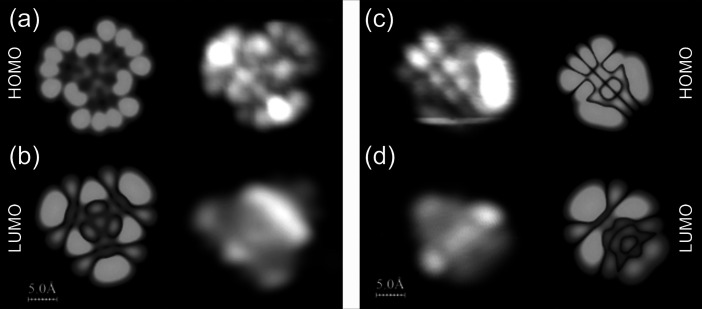
Theoretical simulations of STM images corresponding to HOMO and LUMO orbitals of nanographenes **1** (a, b; left column) and **2** (c, d; right column) combined with high-resolution filled- and empty-state STM images of nanographenes **1** (a, right column: −1.7 V; b, right column: +2.3 V) and **2** (c, left column: −1.5 V; d, left column: +2.2 V) on the TiO_2_(110) surface recorded at voltages that ensure capturing the STS resonances shown in Figure 4a,b. The streaky appearance of the STM image of the filled state of **2** (c) arises from the mobility/instability of the nanographene during high-resolution STM measurements.

In order to verify the reasoning described above, we have applied high-resolution STM imaging combined with theoretical modeling. In general, it is known that filled-state STM imaging on TiO_2_(110) is difficult/unstable;^53,54^ thus, we have not been able to acquire any large-scale images. However, based on multiple attempts, we have recorded high-resolution filled-state STM images of synthesized nanographenes **1** and **2**. The results are visualized in Figure 5. Comparison of experimental filled-state images with the simulated STM images modeled for the gas phase nanographenes **1** and **2** and for voltages corresponding to the HOMO orbital displayed in the top row of Figure 5 provides reasonable agreement. This corroborates the successful synthesis of nanographenes **1** and **2** through the intramolecular cyclodehydrogenation and additionally suggests that the filled-state frontier orbitals of **1** and **2** are not strongly influenced by the substrate.

Contrarily, as the empty-state resonances recorded for **1** and **2** (Figure 4a,b) are located within the range of the surface conduction band, we may expect stronger influence of the substrate on the molecular states and deviations from the gas-phase free molecule picture. Nevertheless, for both **1** and **2**, the agreement between the simulation and the experiment displayed in Figure 5 is reasonable. However, one may notice some minor deviation between the simulated empty-state STM image and experimental one for **2** in the vicinity of the termini, as visualized in Figure 5d. We interpret this as the effect of the interaction with the surface.

At this point, it is worth noting that Sánchez-Sánchez et al.^53^ have reported thermally induced dehydrogenation at TiO_2_(110) followed by subsequent cyclization into chemisorbed nanodomes as well as not controlled intermolecular reactions. However, according to the best of our knowledge, the controlled cyclodehydrogenation into planar nanographenes on semiconductors has not been reported so far.

### On-Surface Synthesis of GNRs

**Figure 6:**
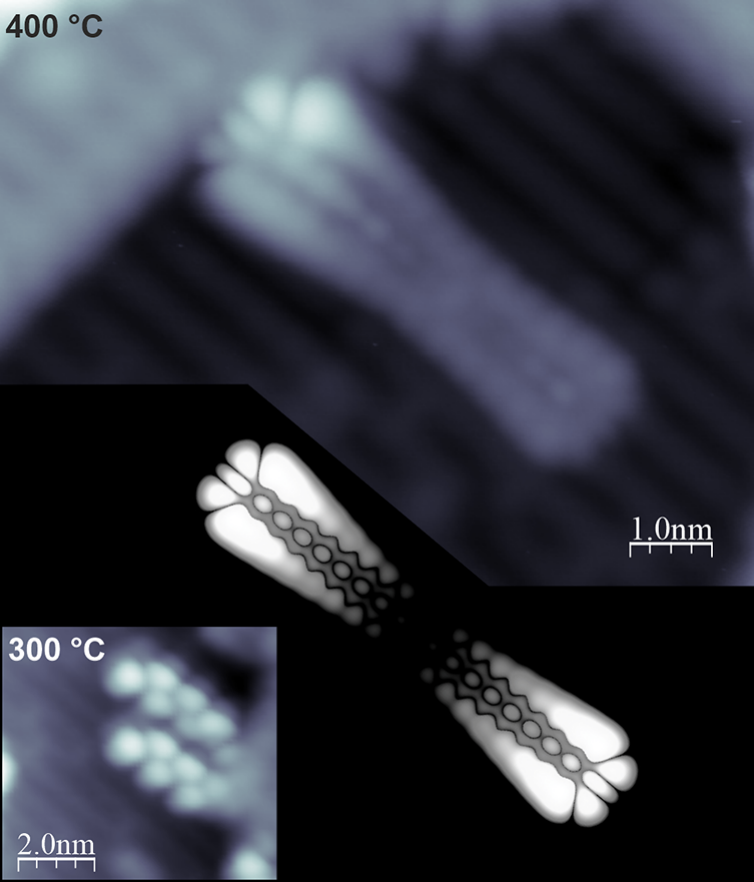
High-resolution STM images of the DBBA based polymers (**7**, left bottom inset, +1.5 V, 7 pA) and 7-AGNR (**3**, top, +1.5 V, 50 pA) fabricated on TiO_2_(110). The bottom panel shows the simulated free-standing STM image of 7-AGNR exhibiting very good agreement with the experiment.

To supplement the successful synthesis of nanographenes, we have attempted the fabrication of GNRs on the TiO_2_(110) surface. In order to achieve the GNR preparation, the two steps of the synthetic process have to be induced, i.e. the transformation of the precursors into polymers followed by the planarization.^11^ As already reported, the dehalogenative C–C coupling could be initiated on the TiO_2_ surface.^33−35^ Based on those reports and the described successful cyclodehydrogenation, we have applied the widely used DBBA precursor (**6**) to generate the archetypic 7-AGNR (**3**). After room temperature deposition, we followed the method introduced by Vasseur et al.^35^ for on-surface polymerization by thermal annealing at 300 °C. The resulting structures are visualized in the inset of Figure 6, where the zigzag lobe pattern, which is characteristic of anthracene-based polymers, is clearly discernible (**7**). Subsequent annealing of the polymers at 400 °C transforms them into 7-AGNR (**3**). The exemplary high-resolution STM image of the generated GNR is shown in Figure 6 together with the image simulated for the free-standing GNR exhibiting very good agreement. This indicates the limited influence of the substrate on the GNR, similarly reported by Kolmer et al. for 7-AGNRs on TiO_2_(011),^47^ and the presence of the edge-states associated with the zigzag termini, which results in the characteristic STM appearance.^55,56^

In previous reports,^35^ it has been demonstrated that the removal of the polymerization byproducts, i.e., bromine, is successfully achieved above 300 °C. Therefore, we could infer that in our experiments the polymerization of DBBA units most likely proceeds in the presence of Br atoms; however, the planarization takes place at higher temperatures (400 °C), suggesting that in the case of 7-AGNR measurements the bromine shall be already removed from the surface.

It is worth noting here that the GNRs synthesized in our experiment usually orient themselves along the surface reconstruction rows and exhibit increased mobility along the rows. In contrast to the GNRs generated through the combination of cyclodehydrodefluorination and tip manipulation on TiO_2_(011)^47^ found exclusively pinned by surface defects, here the GNRs are also distributed on the surface terraces. However, the efficiency of the GNR fabrication is much lower compared to the synthesis of nanographene **1**, which could be interpreted as resulting from the limited efficiency of the polymerization step on the TiO_2_(110) surface (see Supporting Information Figure S4).

## Conclusions

In summary, we have demonstrated the ability to efficiently initiate intramolecular cyclodehydrogenation leading to planar, differently shaped nanographenes and GNRs on TiO_2_(110). The surface assisted synthesis has been confirmed by the high-resolution STM imaging corroborated by STS measurements and theoretical modeling. The combination of experimental and theoretical investigations indicates also that the filled-state frontier orbitals of nanographenes are not significantly disturbed by the substrate. Our findings provide perspectives for the surface-assisted synthesis of a wide range of nanographenes and GNRs on a TiO_2_(110) semiconductor surface. Further studies may also explore the ability to expand the established reaction pathways toward tuning of the product structure and properties, e.g., by inclusion of nonbenzenoid rings or doping units.

## Methods

### Experimental Procedure

The experiments were performed in a multichamber ultrahigh vacuum (UHV) system with the base pressure below 2 × 10^–10^ mbar. As a substrate, single crystal rutile TiO_2_(110)-(1×1) was used (MaTecK GmbH). The surface of the sample was prepared by several cycles of Ar^+^ sputtering at room temperature (15 min) and subsequent AC current annealing at 780 °C for 10 min.^34^ The temperature of the sample during the preparation procedure was monitored by an infrared pyrometer (ε = 36%). The quality of the prepared samples was monitored by STM imaging. The Au(111) monocrystalline samples were prepared by the standard procedure of simultaneous annealing and ion sputtering (Ar^+^ ions). The molecular precursors **4**, **5**, and **6** were deposited from a water-cooled three-cell Kentax GmbH Knudsen cell. The temperature of the deposition was 292, 190, and 160 °C, respectively. For all experiments, the molecular flux was calibrated with the application of a quartz microbalance (1 Hz/5 min). All STM/STS (ScientaOmicron GmbH) measurements were performed at liquid nitrogen temperature (77 K) with Pt–Ir etched tips used as probes. The dI/dV spectra were collected with the use of a lock-in amplifier technique (f = 670 Hz, Amp. = 12 mV). Before switching off the feedback loop and acquisition of the STS data, the tip was stabilized with the voltage bias corresponding to the starting voltage setting of the STS data acquisition (i.e., −2.0 V for the filled-state part of the spectrum and +3.0 V for the empty-state part of the spectrum).

### Image Simulations

The simulated STM images were generated with the codebase of Espeem.^57^ Geometric relaxations were performed using spin-polarized density-functional theory (DFT) using the SIESTA code.^58^ We used a double-ζ polarized (DZP) basis set with orbital radii defined using a 100 meV energy shift, the Perdew–Burke–Ernzerhof (PBE) exchange-correlation potential,^59^ and a real-space grid equivalent to a 200 Ry plane-wave cutoff. Forces were relaxed until forces were smaller than 0.040 eV/Å. Final evaluation was performed with 30 Å of vacuum. To compute the STM images, we followed the surface integration technique of Paz and Soler.^60^ We used the Tersoff-Hamann approximation^61^ assuming a proportionality factor of 1.00 nA·Å^–3^ for the ratio between the local density of states and the current.

## Supporting Information Available

Details of molecular precursor synthesis, additional STM images, and temperature dependent studies of on-surface synthesis of nanographenes **1** and **2** (PDF)
