# Supplementary material for: On-Surface Synthesis of Nanographenes and Graphene Nanoribbons on Titanium Dioxide
Source: ACS Nano. 2023 Jan 24;17(3):2580–7. doi: 10.1021/acsnano.2c10416 (PMC9933590; doi:10.1021/acsnano.2c10416)
Supplement: Supplementary file 1 — nn2c10416_si_001.pdf [file nn2c10416_si_001.pdf]

Supporting information

On-Surface Synthesis of Nanographenes and Graphene Nanoribbons on Titanium Dioxide

Rafal Zuzak,[1] Jesus Castro-Esteban,[2] Mads Engelund,[3] Dolores Pérez,[2] Diego Peña,[2,] Szymon Godlewski[1,]**

[1]Centre for Nanometer-Scale Science and Advanced Materials, NANOSAM, Faculty of Physics, Astronomy and Applied Computer Science, Jagiellonian University, Łojasiewicza 11, PL 30-348 Krakow, Poland

[2]Centro de Investigación en Química Biolóxica e Materiais Moleculares (CiQUS) and Departamento de Química Orgánica, Universidade de Santiago de Compostela, 15782-Santiago de Compostela, Spain

[3]Espeem S.A.R.L. (espeem.com), 12 Cité Franz Leesbierg, L-4206 Esch-sur-Alzette, Luxembourg

1. Synthesis of molecular precursors by solution chemistry

1.1. General methods

All reactions were carried out under argon using oven-dried glassware. TLCs were performed on Merck silica gel 60 F_{254}; chromatograms were visualized with UV light (254 and 360 nm). Flash column chromatography was performed on Merck silica gel 60 (ASTM 230-400 mesh). ^{1}H and ^{13}C NMR were recorded at 300 and 75 MHz (Varian Mercury 300). APCI spectra were determined on a Bruker Microtof instrument. Commercial reagents were purchased from ABCR, GmbH, Aldrich Chemical Co., and were used without further purification. MeCN was purified by a MBraun SPS-800 Solvent Purification System.

Hexapole pentahelicene **4**[1] and 10,10'-dibromo-9,9'-bianthracene (**6**)[2] were prepared following published procedures (Figure S1).

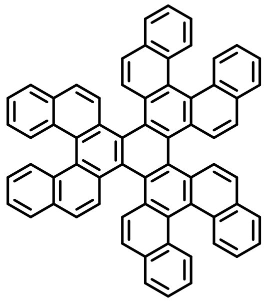

4

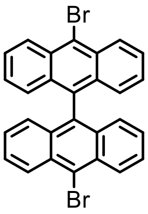

6

*Figure S1. Structure of compounds **4** and **6**.*

1.2. Synthesis of pentahelicene 5.

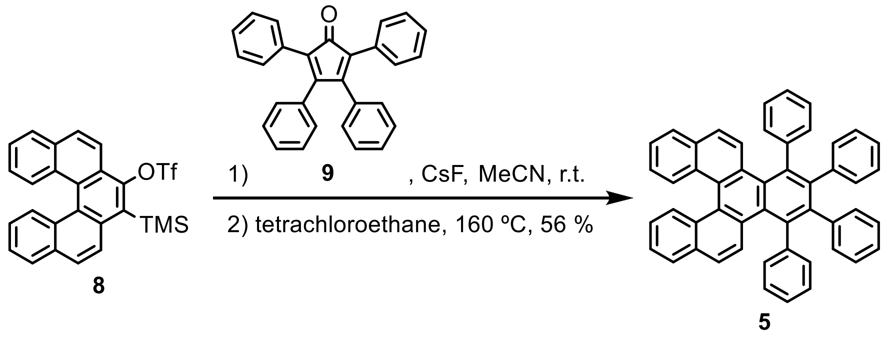

*Figure S2. Synthesis of compound **5**.*

To a solution of triflate **8**[1] (50.0 mg, 0.100 mmol) and compound **9** (46.1 mg, 0.120 mmol) in MeCN (2 mL), anhydrous CsF (91.1 mg, 0.600 mmol) was added. Then, the mixture was stirred 10 h under argon atmosphere at room temperature. After this time, the reaction mixture was concentrated under reduced pressure, the resulting mixture was dissolved in tetrachloroethane (5 mL) and heated to reflux (160 ºC) for 12 h. Then, the mixture was concentrated under reduced pressure and the crude was purified by column chromatography (SiO_2, CH_2Cl_2:hexane, 2:8) affording compound **5** (35.4 mg, 56 %) as a pale yellow solid.

^{1}H RMN (298 K, 300 MHz, $CDCl_3$) δ: 8.11 (d, *J* = 8.6 Hz, 2H), 7.78 (d, *J* = 8.1 Hz, 2H), 7.67 (d, *J* = 9.1 Hz, 2H), 7.53 (d, *J* = 9.1 Hz, 2H), 7.43 (t, *J* = 7.4 Hz, 2H), 7.27 – 7.11 (m, 12H), 6.94 (m, 6H), 6.84 (m, 2H), 6.61 (d, *J* = 6.6 Hz, 2H) ppm. **^{13}C RMN** (298 K, 75 MHz, $CDCl_3$) δ: 142.52 (2xC), 140.52 (2xC), 140.40 (2xC), 136.48 (2xC), 132.91 (2xCH), 132.23 (4xCH), 132.21 (2xC), 131.76 (2xC), 131.24 (2xCH), 130.89 (2xC), 130.78 (2xC), 129.63 (2xCH), 128.56 (2xCH), 127.82 (2xCH), 127.74 (2xCH), 126.95 (2xCH), 126.86 (2xC), 126.78 (4xCH), 126.66 (2xCH), 126.00 (2xCH), 125.50 (2xCH), 125.13 (2xCH), 124.79 (2xCH) ppm. **HRMS** (APCI) for $C_{50}H_{32}$, calculated: 633.2580, found: 633.2577.

1.3. ^{1}H, DEPT and ^{13}C NMR spectra of pentahelicene 5.

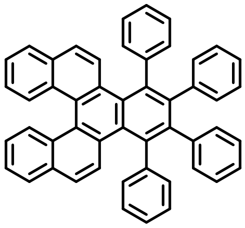

5

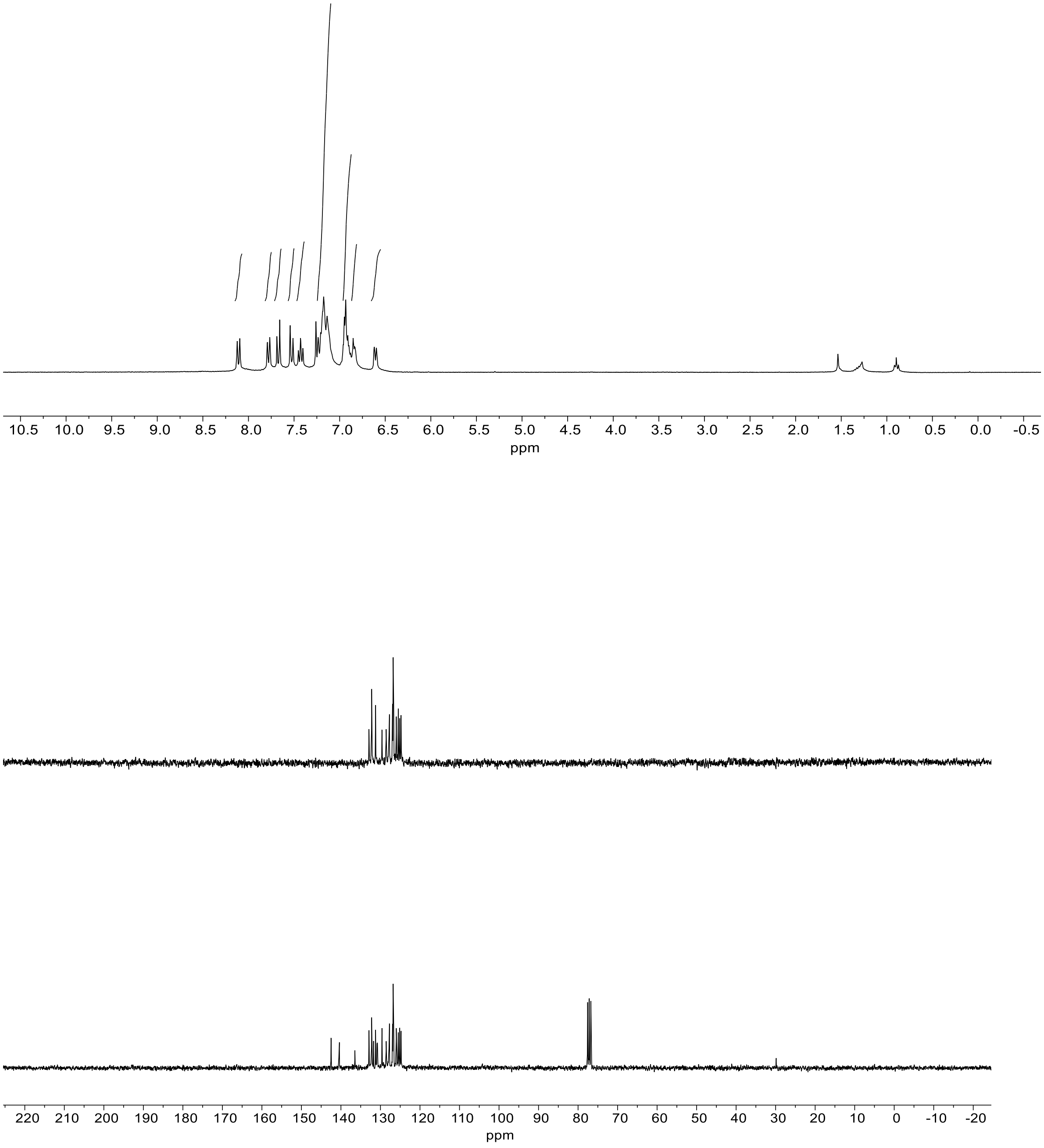

*Figure S3. ^{1}H (top), DEPT (middle) and ^{13}C (bottom) NMR spectra of pentahelicene **5**.*

1.4. Additional STM images.

Figure S4 shows the STM image of the TiO_2 (110) surface with 7-AGNRs and smaller units synthesized by the combination of Ullmann-like polymerization and surface assisted cyclodehydrogenation. The synthesis of GNRs is limited by the low efficiency of the polymerization step.

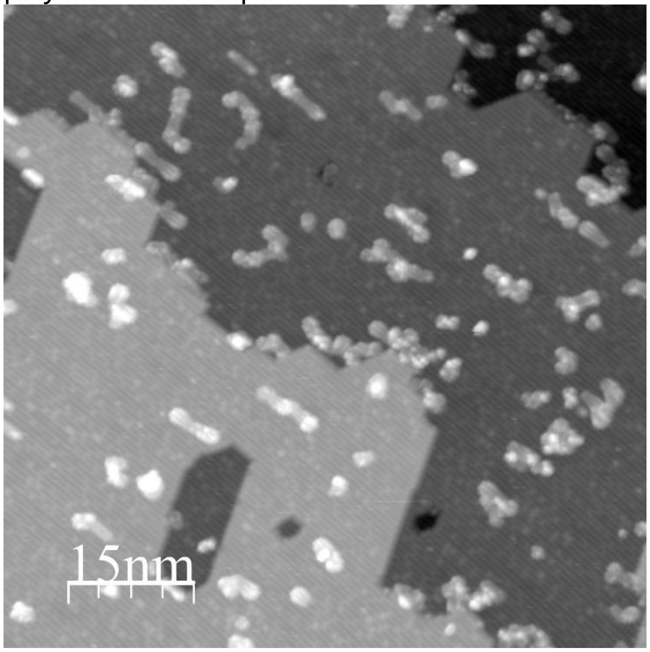

*Figure S4. STM image of 7-AGNRs (**7**) and smaller units synthesized from DBBA (**6**) precursors on the TiO_2 (110), tunneling current: 50 pA; bias voltage: +1.5 V.*

Figure S5 shows the STM image of the precursors 4 after deposition on the TiO_2 (110) surface.

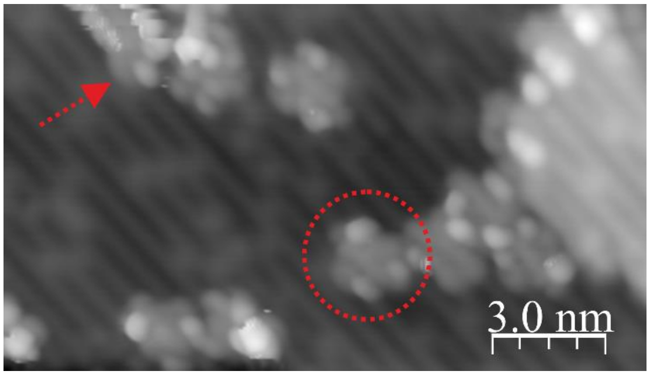

*Figure S5. STM image of precursors 4 after deposition on the TiO_2 (110),red arrow indicates the mobile precursor **4**, red dashed circle marks exemplary precursor **4**; tunneling current: 50 pA; bias voltage: +1.5 V.*

1.5. Temperature dependent studies of on-surface synthesis of nanographenes 1 and 2.

In order to estimate the efficiency of the cyclodehydrogenation reaction we have performed annealing of the samples after deposition of precursors. The processes were performed for 20 minutes at 300 °C, 350 °C and 400 °C. In all cases the deposition of the starting material was performed in an identical manner to ensure comparable amount of precursors.

1.5.1 transformation 4 → 1.

After annealing we have noted the following findings (calculated in each case from 450-550 molecules):

- annealing at 300 °C, only single nanoflakes **1** are found - less than 1% of precursors **4** transformed into nanoflakes **1**
- annealing at 350 °C, approximately 25% of precursors **4** found transformed into nanoflakes **1**
- annealing at 400 °C, more than 99% of precursors **4** are transformed into nanoflakes **1** (only single objects that may correspond to not fully transformed precursors)

In all above mentioned experiments we do not observe differences in the coverage of molecules, which indicates that precursors **4** do not desorb up to 400 °C further supported by the formation of a closed layer of nanographenes 1.
The above described observations are illustrated below in Figure S6 by STM images acquired for the samples with precursors **4** annealed to 300, 350 and 400 °C.

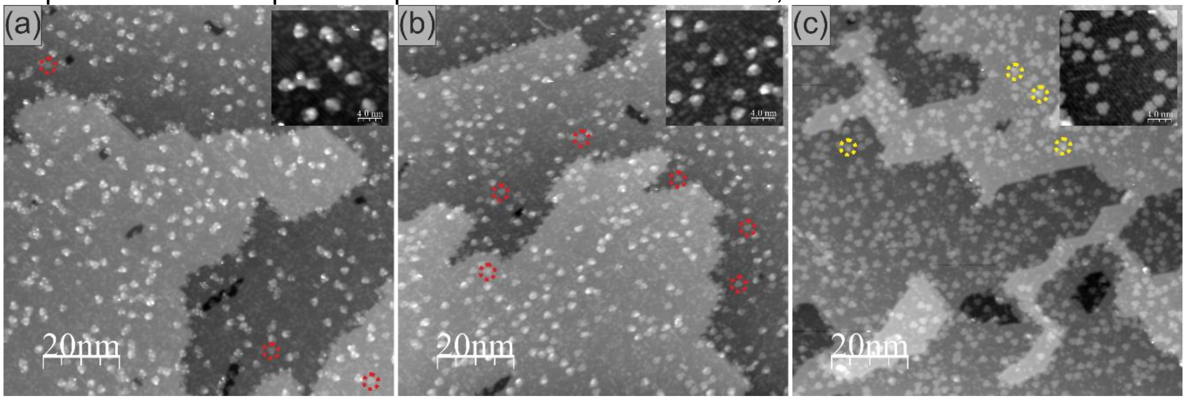

*Figure S6. STM images of precursors **4** after deposition on the TiO_2 (110) and annealing for 20 minutes at: (a) 300 °C, (b) 350 °C, (c) 400 °C; red dashed circles indicate examples of nanographenes **1**, yellow dashed circles mark species that ma correspond not to fully planar nanographenes **1**;insets show zoomed images; tunneling current: 25 pA; bias voltage: +1.2 V.*

1.5.2 transformation 5 → 2.

In case of precursors **5** the detailed quantitative analysis is hampered due to the following factors:

- precursors **5** tend to form disordered molecular assemblies precluding from doubtless identification of single species
- the comparison of STM images acquired for samples annealed to 300 °C and 350 °C suggests that precursors 5 start to desorb from the surface already below 350 °C
- in all cases (after annealing to 300, 350 and 400 °C) a fraction of molecular species could be found in assemblies and at steps making their doubtless identification/differentiation extremely challenging

Due to the above described observations the estimated efficiency is subjected to significant error.

Based on the temperature study of precursors **5** we may note the following conclusions:

- annealing at 300 °C, we have not observed any nanoflakes **2**
- annealing at 350 °C, below 5% of molecules on the surface could be identified as nanoflakes **2**, we note here that the detailed identification of the molecules in assemblies and at steps is hampered
- annealing at 400 °C, we estimate that 60 (±10) % of observed molecular species could be identified as nanoflakes **2** (calculated from approximately 400 molecules), we note here that less than 25% of initially deposited precursors **5** could be still found on the surface (more than 75% of precursors desorbed). This gives the approximate estimation of the overall efficiency of the conversion **5** → **2** at the level of 15 (±3) %.

The above described experiments are illustrated by STM images in Figure S7.

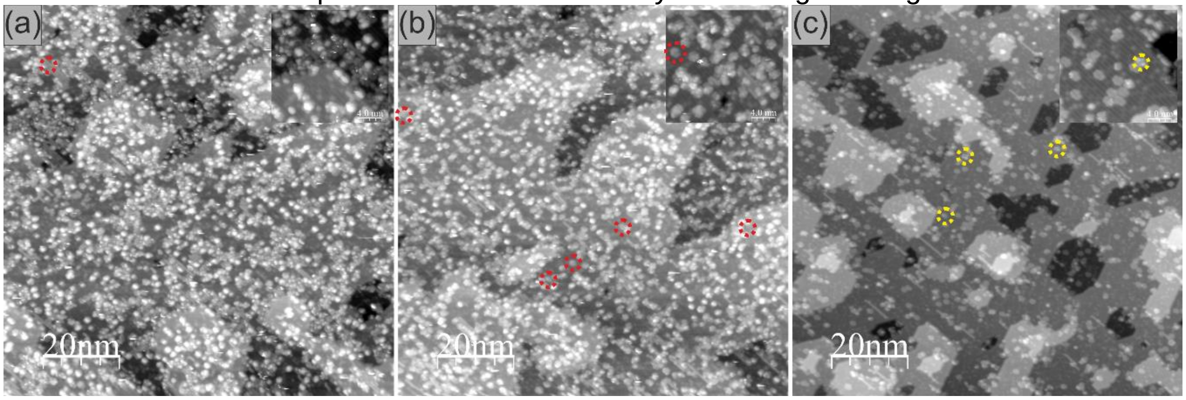

*Figure S7. STM images of precursors **5** after deposition on the TiO_2 (110) and annealing for 20 minutes at: (a) 300 °C, (b) 350 °C, (c) 400 °C; red dashed circles indicate examples of nanographenes **2**, yellow dashed circles mark species that ma correspond not to fully planar nanographenes **2**;insets show zoomed images; tunneling current: 25 pA; bias voltage: +1.2 V.*

1.6 Hexaphenylbenzene.

We have attempted the synthesis of hexabenzocoronene molecules through the cyclodehydrogenation between neighboring phenyl rings of hexaphenylbenzene precursors (purchased from Sigma Aldrich). The target compound has not been achieved, because the precursors desorb at the temperature below 350 °C. The STM images of the self-assembled precursors after annealing to 300 °C and the surface after annealing to 350 °C are shown in Figure S8.

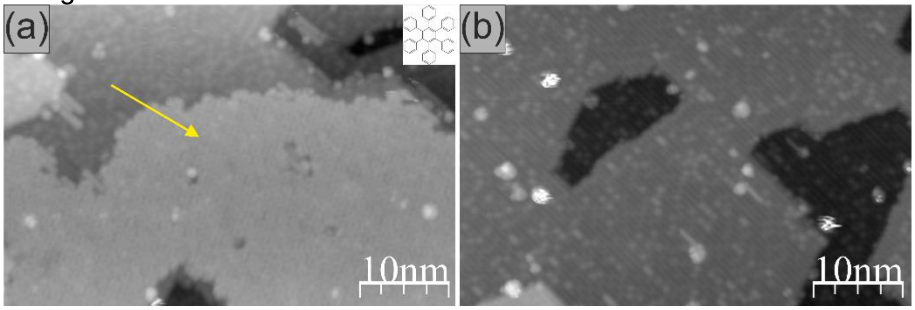

Figure S8. STM images of hexaphenylbenzene precursors (a) after annealing at 300 °C and (b) annealing at 350 °C (precursors desorbed) on the TiO_2 (110);yellow arrow in (a) indicates the self-

assembled layer of hexaphenylbenzenes; tunneling current: 15 pA (a), 25 pA (b), bias voltage: +1.3 V.

1.7 References.

[1] R. Zuzak, J. Castro-Esteban, P. Brandimarte, M. Engelund, A. Cobas, P. Piatkowski, M. Kolmer, D. Pérez, E. Guitián, M. Szymonski, D. Sánchez-Portal, S. Godlewski, D. Peña, *Chem. Commun.*, **2018**, *54*, 10256.

[2] J. Cai, P. Ruffieux, R. Jaafar, M. Bieri, T. Braun, S. Blankenburg, M. Muoth, A. P. Seitsonen, M. Saleh, X. Feng, K. Müllen, R. Fasel, *Nature*, **2010**, *466*, 470.
